# Drug–target interaction prediction using unifying of graph regularized nuclear norm with bilinear factorization

**DOI:** 10.1186/s12859-021-04464-2

**Published:** 2021-11-17

**Authors:** Ali Ghanbari Sorkhi, Zahra Abbasi, Majid Iranpour Mobarakeh, Jamshid Pirgazi

**Affiliations:** 1 Faculty of Electrical and Computer Engineering, University of Science and Technology of Mazandaran, P.O. Box, 48518-78195 Behshahr, Iran; 2 School of Medicine, Faculty of Medical Biotechnology, Shahroud University of Medical Sciences, Shahroud, Iran; 3 Faculty of Computer Engineering and IT, Payam Noor University, Tehran, Iran

**Keywords:** Drug–target interaction, Computational prediction, Low-rank interaction, Drug discovery

## Abstract

**Background:**

Wet-lab experiments for identification of interactions between drugs and target proteins are time-consuming, costly and labor-intensive. The use of computational prediction of drug–target interactions (DTIs), which is one of the significant points in drug discovery, has been considered by many researchers in recent years. It also reduces the search space of interactions by proposing potential interaction candidates.

**Results:**

In this paper, a new approach based on unifying matrix factorization and nuclear norm minimization is proposed to find a low-rank interaction. In this combined method, to solve the low-rank matrix approximation, the terms in the DTI problem are used in such a way that the nuclear norm regularized problem is optimized by a bilinear factorization based on Rank-Restricted Soft Singular Value Decomposition (RRSSVD). In the proposed method, adjacencies between drugs and targets are encoded by graphs. Drug–target interaction, drug-drug similarity, target-target, and combination of similarities have also been used as input.

**Conclusions:**

The proposed method is evaluated on four benchmark datasets known as Enzymes (E), Ion channels (ICs), G protein-coupled receptors (GPCRs) and nuclear receptors (NRs) based on AUC, AUPR, and time measure. The results show an improvement in the performance of the proposed method compared to the state-of-the-art techniques.

## Background

The study of DTIs has been attracted many researchers’ attention in the field of pharmaceutical science in recent years [1–4]. In this regard, many efforts have been made to investigate drug repositioning as well as the discovery of the interaction between new targets and existing drugs. DTI means binding a drug to a target location, that leads to a change in its behavior or function. On the other hand, the identification of DTIs minimizes the adverse side effects of drugs [1].

Performing wet-lab experiments is a significant challenge in terms of cost, time and effort [5]. In this regard, Computational Prediction (CP) methods have been used in recent years [6]. In addition, there is ample evidence of Disease-Associated Microbes (DAM) as well as Long non-coding RNA (lncRNA)-Disease Associations (LDA) [7, 8]. Using traditional approaches of experiments to confirm these connections often requires a great deal of materials and time which are expected computational methods to be used to predict these associations. Many of these algorithms use profile-based methods (for example, NCPLP [7] in ADM and BLM-NPAI [8] in LDA) to predict these associations.

Despite the synthesis of many compounds, their target profiles and drug effects are still unidentified. Besides, there is no cure for many diseases and many new diseases are introduced each year. Therefore, much information has been gathered about various compound properties, features, responses and target proteins by researchers. The emergence of a large dataset has led to the use of CP with problems such as high dimensional, complex data, which indicates the need for efficient and robust algorithms in DTIs.

Computational methods for DTIs have been used by state-of-the-art researchers. Generally, three main categories can be introduced for computational methods in this application [1]. In the first category, the concept of that similar molecules tend to share similar properties and usually bind similar proteins, is used which these methods are called ligand-based approaches [6, 9, 10]. This approach predicts interactions using similarities between identical protein ligands. Since these ligand-based methods do not use sequence information of the proteins for prediction, it is possible that a novel interaction restricts to link between known ligands and protein families. On the other hand, the performance of these methods is highly dependent on known ligands, and if these ligands are low for a candidate protein, the performance of these methods is drastically reduced [1, 11].

In the second category, the 3D structures of drugs and proteins are used by a simulation to determine the interaction, known as docking approaches. The main problem is that the 3D structure of some proteins is not known [12, 13]. The third category is chemogenomic-based methods that uses information about drugs and targets simultaneously. This method has been considered by many researchers in recent years; furthermore, it can be used in a broad biological data, which are also used for the prediction of data from process information such as chemical structure graphs and genomic sequences for the drugs and targets from both sides of the drug and target simultaneously [14]. For this purpose, biological information that is available in public datasets can be used. This general method can be divided into two categories: Feature-based and similarity-based methods [15]. In the feature-based method, a supervised machine learning technique is used. In fact, in this method, feature vectors use sets of drug–target pairs with class labels that indicate the presence of interaction (positive instance) or no interaction (negative example). Also, it should be noted that negative samples are samples without non-interactions or unknown drug–target interactions [16–18]. In similarity-based methods, two matrices of similarity related to the drug and similarity of the target along with the interaction matrix are used which represent the interaction between the drug and the target [19–21]. These similarities are usually created for the drug by chemical structures and for the target by protein sequence alignment. Similarity-based methods have several apparent advantages [22]:

1. Feature-based approaches require a feature extraction or selection process which is complex and challenging, while similarity-based techniques do not require this process.
2. Computing similarity measures have already been expanded and used extensively such as chemical structure similarity for drugs and genomic sequence similarity for targets.
3. Similarity-based approaches can provide better performance in prediction since directly related to kernel methods.
4. Similarity matrices represent chemical space and genomic space derived from the relationships between drugs and genes, respectively.

These advantages demonstrate the superiority of similarity-based approaches over other approaches. Adjacency matrices are commonly used to represent drug-drug and target-target similarity.

Another point of view that can be introduced to categorize the available methods in DTI is methods which includes classification, network inference and matrix factorization groups. Classification-based models are divided into Local Classification Model (LCM) [23, 24] and Global Classification Model (GCM) [25, 26]. It is difficult to diagnose drugs (resp. targets) that interact with the same target (resp. drug), so the LCM is not able to show a link between targets or drugs [27]. Also, GCM cannot show the relationship between targets or drugs due to the complexity of similarity calculations based on the tensor product or high dimensional concatenate feature vectors. Overall, these models do not easily capture the underlying structure among drug–target pairs [28].

In recent years, many types of researches have been done based on deep learning in DTI [29–31]. A comprehensive deep learning library called DeepPurpose has been introduced for DTI prediction [29]. This library includes the implementation of 15 compound and protein encoders and more than 50 neural architectures with other beneficial features in DTI.

Convolution Neural Networks (CNNs) were used to obtain 1-dimensional representations of protein sequences (amino acid sequences) and simplified molecular input line-entry system (SMILES) compounds in [30]. The extraction features were claimed to show an appropriate representation of local dependencies or patterns and serve as a suitable input for a fully connected neural network (FCNN) for the binary classifier. The results show that the use of CNNs to obtain data display, as an alternative to traditional descriptors, improves performance in DTI.

A deep learning model based on DeepLSTM was developed to predict DTI in [31]. Position-Specific Scoring Matrices (PSSM) and Legendre Moment (LM) were used to extract the evolutionary features of proteins. The Sparse Principal Component Analysis (SPCA) was then used to compress the features of drugs and proteins in a uniform vector space.

It should also be noted that the use of deep learning also faces with similar problems; on the other hand, the use of deep learning has a large dataset for training, which unfortunately in these applications, providing data is expensive and time-consuming.

Interactions between drugs and targets show a significant relationship that is represented by a bi-partite network [32]. The information in this network is taken from drug–target interactions. A bi-partite network is based on network inference (e.g., NBI [19]) which has transformed DTI prediction to link prediction between graph nodes. Two-step resource allocation is used by NBI to infer the potential links between nodes. Although, it just depends on the local or the first-order topology of nodes, it tends to completely bias the high-degree nodes [26]. In addition, NBI cannot predict the interaction between the target-drug pair without known accessible pathways in the network [32]. The heterogeneous network is a promising model. This network is made by a DTI network and two other networks which is produced by drug similarities and target similarities, respectively [28, 33].

The models based on matrix factorization, such as BMF2K [34], CMF [35], NRLMF [36] are good models to obtain structural information between drug–target interactions. Accordingly, drugs and targets are planned to a common low-rank feature space according to the drug similarity matrix and the target similarity matrix [28]. Two networks (dual-network L_2,1_-collaborative matrix factorization) have been proposed to predict Drug-Disease Interactions (DDI), called the L_2,1_-network matrix factor [37]. In this method, to achieve better results, the Gaussian interaction profile kernels and L_2,1_-norm are presented. Moreover, the network similarities of drugs and diseases are combined with their chemical and semantic similarities. In order to identify potential links in biomedical bi-partite networks, a method called graph regularized generalized matrix factorization (GRGMF) is proposed to predict links [38]. For this purpose, a matrix factorization model is formulated to use latent patterns behind observed links. It is claimed that the results showed an improvement in the proposed method.

In this regard, factorization approaches can be used to predict DTIs [21]. In general, based on the reported experiences [6], matrix factorization methods have achieved the best results in DTI. Since there are few factors for DTI and these latent factors characterize drugs and targets, the DTI matrix can be converted into a latent factor matrix of drug and target. The DTI matrix is of low-rank which can be solved using matrix factorization. Matrix factorization is a bi-linear non-convex problem that there are no convergence guarantees [11]. Nuclear Norm Minimization (NNM) based methods have also been proposed to improve it. By shrinking all Singular Values (SVs) uniformly, the NNM is usually used to estimate the matrix rank. Despite the precise physical meanings of SVs, NNM cannot accurately estimate the matrix rank.

In this paper, unifying matrix factorization and NNM approaches combined with graph regularization penalties are proposed, which will be described in detail in the following sections. It should be noted that similarly to Mongia et al. study [11], a similarity matrix is used in this proposed method.

The strengths of the proposed method are as follows:

- Unifying nuclear norm with bilinear factorization is presented based on the similarity of drug-drug and target-target, which has caused the advantages of both methods to be combined.
- Rank-Restricted Soft Singular Value Decomposition method is used to optimize the nuclear norm minimization in the DTI problem.
- The performance of the proposed method based on AUC and AUPR measures had the best performance and also the results were suitable in datasets with different features.
- The time complexity of the running time is $O\left( {r^{3} + n^{b} \log \left( n \right)} \right)$ for each iteration in the proposed algorithm. This complexity is polynomial, which has performed better than other new methods.
- The proposed method can be widely used in other applications of CP. Using the proposed method does not require a complex process.

The rest of the paper is organized as follows: In “Methods” section, the proposed method that includes a novel algorithm for DTI is introduced. The experimental setup and results, that include the introduction of datasets, evaluation criteria and comparison of methods, are presented in “Discussion” section. Finally, the conclusion and discussion are presented in “Conclusion” section.

## Methods

The interaction between the target and the drug is shown by the adjacency matrix X, where drugs are presented in rows and targets are presented in columns. The value of the matrix represents the interactions. Since all DTIs are not known, this matrix is partially observed. This is expressed as follow:

1 $$Y = P\left( X \right)$$

In Eq. (1), *P* is a sub-sampling operator which in this binary sampling matrix the value of 1 means that there is a known interaction and 0 means otherwise. *Y* is an available partially sampled DTI matrix. The purpose of this equation is to estimate the matrix *X* given *Y* and known *P.* *X* is a low-rank that needs to be recovered. Equation (2) can be used for this purpose.

2 $$\begin{array}{*{20}c} {\begin{array}{*{20}c} {min} \\ X \\ \end{array} } & {rank\left( X \right) \; such\; that\; Y = P\left( X \right)} \\ \end{array}$$

Low-Rank Matrix Approximation (LRMA) has been used in many practical cases that have low rank properties, so in recent years it has attracted considerable interest in different areas, such as computer vision and machine learning [39–42]. In general, LRMA methods are divided into two categories, the low rank matrix factorization (LRMF) [43–46] and the rank minimization methods [47–49]. The purpose of the LRMF concerning the input matrix Y is to factorize it to the product of two low rank matrices that can be used to reconstruct the low rank matrix X with exceptional fidelity. A variety of LRMF-based methods, such as classical Singular Value Decomposition (SVD) under '${\mathcal{L}}_{2} - {\text{norm}}$ '[50, 51], robust LRMF methods under ‘${\mathcal{L}}_{1} - {\text{norm}}$’ [52, 53] and other probabilistic methods have been proposed [54, 55]. The problem of Low rank models for recovering a rank-k matrix Z can be expressed by minimizing Eq. (3).

3 $$\begin{array}{*{20}c} {\begin{array}{*{20}c} {min} \\ Z \\ \end{array} } & {f\left( {Y - Z} \right) \; subject \;to \;rank\left( Z \right) = k} \\ \end{array}$$

where f(.) defines a loss function.The rank limitation in Eq. (3) has typically been imposed by a factorization ${\text{Z}} = {\text{AB}}^{{\text{T}}}$, as

4 $$\begin{array}{*{20}c} {\begin{array}{*{20}c} {min} \\ {A,B} \\ \end{array} } & {f\left( {Y - AB^{T} } \right) } \\ \end{array}$$

based on its intractability. It has been proven that when the loss function is the Least Squares (LS) loss, i.e., ${\text{f}}\left( {{\text{Y}} - {\text{AB}}^{{\text{T}}} } \right) = \left\| {{\text{Y}} - {\text{AB}}^{{\text{T}}} } \right\|_{{\text{F}}}^{2}$, then Eq. (4) does not have local minima and a closed form solution can be obtained via the SVD of Y [56]. One of the disadvantages of this factorization approach is highly susceptibility of the LS loss to outliers and the presence of missing data in Y results in local minima. Factorization with missing data is a NP-Hard problem [57], while outliers can be addressed with robust loss functions [58, 59].

In DTI, matrix X can be converted into two matrices as follows:

5 $${\text{ X}}_{{{\text{M}} \times {\text{N}}}} = {\text{A}}_{{{\text{M}} \times {\text{k}}}} {\text{B}}_{{{\text{k}} \times {\text{N}}}} ,{\text{ k}} < < \left( {{\text{m}},{\text{n}}} \right)$$

Here M and N are the numbers of drugs and targets, respectively and k is the presumed rank of the matrix. The Eq. (4) for DTI is expressed as Eq. (6).

6 $${ }\begin{array}{*{20}c} {\begin{array}{*{20}c} {{\text{min}}} \\ {{\text{A}},{\text{B}}} \\ \end{array} } & {{\text{f}}\left( {{\text{Y}} - {\text{P}}({\text{AB}}^{{\text{T}}} )} \right){ }} \\ \end{array} { }$$

As mentioned earlier, the second category of LRMA methods is based on rank minimization. These methods by setting an additional rank constraint on the estimated matrix can reconstruct the data matrix. Direct rank minimization is challenging to solve because these are NP-hard. To solve this type of problem, the NNM methodology is used. In this methodology, the problem is generally solved by replacement minimizing the nuclear norm of the estimated matrix that is a convex relaxation of minimizing the matrix rank. $\left\| X \right\|_{*}$ is the nuclear norm of matrix *X*. For example, $\left\| X \right\|_{*} = \mathop \sum \limits_{i} \sigma_{i}$ is the nuclear norm which is the sum of its SV that $\sigma_{i}$ represents the i-th SV of the matrix *X*. NNM attempts to recover matrix *X*, actual low rank, by minimizing $\left\| X \right\|_{*}$ from degraded observation matrix *Y*. In recent years, NNM-based methods have been used in many applications such as video denoising [60], background extraction [61], data recovery [62] and subspace clustering [63, 64]. The matrix rank can be recovered under the conditions of the limited and theoretic warranty. However, in some applications, it acts the various rank components equally, and therefore it cannot be precise enough to estimate the matrix rank. Thus several methods have been proposed to improve NNM performance [11, 65].

For noisy input, by solving the NNM problem, the inherent low rank reconstruction can be achieved with a high probability. Also, the Nuclear Norm Proximal (NNP) is also represented by the following equation:

7 $$\begin{array}{*{20}c} {\begin{array}{*{20}c} {min} \\ Z \\ \end{array} } & {f\left( {Y - Z} \right) + \lambda \left\| Z \right\|_{*} } \\ \end{array}$$

By using a soft threshold process on the SV of the observation matrix, it can be easily solved in closed form:

8 $$\hat{X} = US_{{\frac{\lambda }{2}}} \left( {\Sigma } \right)V^{T}$$

In this equation, ${\text{Y}} = {\text{U}}\Sigma {\text{V}}^{{\text{T}}}$ is the SVD of Y, the soft thresholding function on diagonal matrix ${\Sigma }$ with parameter $\frac{\lambda }{2}$ is indicated by ${\text{S}}_{{\frac{\lambda }{2}}} \left( \Sigma \right)$. For each diagonal element $\Sigma_{{{\text{ii}}}}$ in $\Sigma$, there is:

9 $$S_{{\frac{\lambda }{2}}} \left( \Sigma \right)_{ii} = max\left( {\Sigma_{ii} - \frac{\lambda }{2},0} \right)$$

λ is considered as a trade-off parameter between the loss function and the low-rank regularization, that is created through the nuclear norm. These models have generalized the use of low-rank compared to many applications, where Z is low rank but has no a priori [40, 66].

In addition to having convexity and theoretic guidance of the λ [40], these models also have multiple drawbacks.

To show how to create a determined rank in Z, by setting λ [67], Z has a predetermined rank. It usually gives more undesirable results than its direct usage in Eq. (2). Additionally, the “kernel trick” cannot be used because access to the factorization of Z in Eq. (3) is not available. Also, Eq. (3) is a Semidefinite Program (SDP) and Off-theshelf SDP optimizers are suitable for low-middle dimensional optimization (i.e., hundreds of variables) and are not amenable for large scale datasets with the high dimension.

To deal with the limitations, this paper uses a robust method called Rank-Restricted Soft SVD (RRSSVD) based on Hastie et al. study [68] for the DTIs. In the following, we will describe this method in this application. Based on [69], the nuclear norm can be expressed as follows:

10 $$\left\| X \right\|_{*} = {}_{A,B}^{min} \frac{1}{2}\left( {\left\| A \right\|_{F}^{2} + \left\| B \right\|_{F}^{2} } \right) {\text{subject}}\;{\text{ to}}\; X = AB^{T}$$

In this section, the relationship between factorization and nuclear norm approaches is used based on the method presented in [67], that bridges the gap between two methods is presented in Eq. (11).

11 $$\begin{array}{*{20}c} {\begin{array}{*{20}c} {min} \\ {A,B} \\ \end{array} } & {f\left( {Y - AB^{T} } \right) + \frac{\lambda }{2}\left( {\left\| A \right\|_{F}^{2} + \left\| B \right\|_{F}^{2} } \right) } \\ \end{array}$$

Equation (11) for the DTI problem can be expressed as follows:

12 $$\begin{array}{*{20}c} {\begin{array}{*{20}c} {min} \\ {A,B} \\ \end{array} } & {\left\| {Y - P(AB^{T} )} \right\|_{F}^{2} + \frac{\lambda }{2}\left( {\left\| A \right\|_{F}^{2} + \left\| B \right\|_{F}^{2} } \right) } \\ \end{array}$$

In this paper, Eq. (12) is used to solve Eq. (13).

13 $$\begin{array}{*{20}c} {\begin{array}{*{20}c} {min} \\ Z \\ \end{array} } & {\left\| {Y - P\left( Z \right))} \right\|_{F}^{2} + \lambda \left\| Z \right\|_{*} } \\ \end{array}$$

To solve the Eq. (13), Algorithm 1 is used based on the RRSSVD method in DTI prediction. In this algorithm, theorems 1 and 2 [68] are used for DTI.

### Theorem 1

For the optimization problem (14), where Y_m×n_ is a fully observed matrix and $0 < {\text{r}} \le {\text{min}}\left( {{\text{m}},{\text{n}}} \right)$.

14 $$\begin{array}{*{20}c} {\begin{array}{*{20}c} {min} \\ {Z:rank\left( Z \right) \le r} \\ \end{array} } & {F_{\lambda } \left( Z \right): = \frac{1}{2}\left\| {Y - Z} \right\|_{F}^{2} + \lambda \left\| Z \right\|_{*} } \\ \end{array}$$

a solution provided by

15 $$\tilde{Z} = U_{r} S_{\lambda } \left( {D_{r} } \right)V_{r}^{T}$$

where the rank-r SVD of Y is ${\text{U}}_{{\text{r}}} {\text{D}}_{{\text{r}}} {\text{V}}_{{\text{r}}}^{{\text{T}}}$ and ${\text{S}}_{\lambda } \left( {{\text{D}}_{{\text{r}}} } \right) = {\text{diag}}\left[ {\left( {\sigma_{1} - \lambda } \right)_{ + } , \ldots ,\left( {\sigma_{{\text{r}}} - \lambda } \right)_{ + } } \right]$.

### Theorem 2

For the optimization problem (14), where $Y_{m \times n}$ is a fully observed matrix and $0 < r \le min\left( {m,n} \right)$.

16 $$\begin{array}{*{20}c} {\begin{array}{*{20}c} {min} \\ {A_{{m \times {\text{r}}}} ,B_{n \times r} } \\ \end{array} } & {\frac{1}{2}\left\| {Y - AB^{T} } \right\|_{F}^{2} + \frac{\lambda }{2}\left( {\left\| A \right\|_{F}^{2} + \left\| B \right\|_{F}^{2} } \right) } \\ \end{array}$$

a solution provided by $\tilde{A} = U_{r} S_{\lambda } \left( {D_{r} } \right)^{\frac{1}{2}}$ and $\tilde{B} = V_{r} S_{\lambda } \left( {D_{r} } \right)^{\frac{1}{2}}$, and all solutions satisfy $\tilde{A}\tilde{B}^{{\text{T}}} = \tilde{Z}$, where $\tilde{Z}$ is as given in (15).

Finally, it should be noted that relative change in the Frobenius norm has been used to check convergence. Equation (17) is used to calculate it. This equation is based on a pair of iterates ${ }\left( {{\text{U}},{\text{D}}^{2} ,{\text{V}}} \right){ }\left( {{\text{old}}} \right)$ and ${ }\left( {\tilde{U},\tilde{D}^{2} ,\tilde{V}} \right){ }\left( {{\text{new}}} \right)$.

17 $$\nabla F = \frac{{\left\| {UD^{2} V^{T} - \tilde{U}\tilde{D}^{2} \tilde{V}^{T} } \right\|_{F}^{2} }}{{\left\| {UD^{2} V^{T} } \right\|_{F}^{2} }} = \frac{{tr\left( {D^{4} } \right) + tr\left( {\tilde{D}^{4} } \right) - 2tr\left( {D^{2} U^{T} \tilde{U}\tilde{D}^{2} \tilde{V}^{T} V} \right)}}{{tr\left( {D^{4} } \right)}}$$

By this algorithm, $\tilde{Z} = \tilde{A}\tilde{B}^{T}$ is considered as the output.
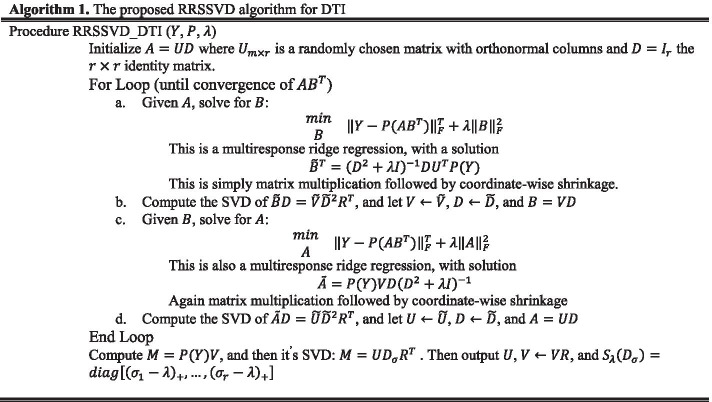


As mentioned in this paper, the adjacency matrix is used that represents the interaction matrix between targets and drugs. In this matrix, if there is a known interaction between the drug (dt) and the target ($t_{j}$), the value is 1 and otherwise the value is zero. In this article, in addition to the interaction matrix, a drug similarity matrix ($S_{d}$) and a target similarity matrix ($S_{t}$) are used. With the number of sub-structures shared in the chemical structure between the two drugs, the SIMCOMP introduced in [70] is used. In fact, $S_{d}$ indicates the similarity of the chemical structure of the drug pair. Similarly, $S_{t}$ represents the degree of similarity between the two proteins, which is calculated from the similarity of the genome sequence based on the amino acid sequence of the target protein. It should be noted that normalized Smith-Waterman [71] has been used to calculate this.

In addition to the use of the similarity matrix introduced, there are four other types of similarity matrices such as Cosine ($S^{cos}$), Correlation ($S^{cor}$), Hamming ($S^{ham}$) and Jaccard ($S^{jac}$) which are used to predict DTI [11]. This paper also uses five similarity matrices calculated using the drug–target interaction matrix. The similarity matrices are used for DTI prediction, which by this method; Eq. (13) is expressed as follows:

18 $$\begin{array}{*{20}c} {\begin{array}{*{20}c} {min} \\ Z \\ \end{array} } & {\left\| {Y - P\left( Z \right))} \right\|_{F}^{2} + \lambda \left\| Z \right\|_{*} + \alpha_{1} Tr\left( {Z^{T} \mathop \sum \limits_{i = 1}^{nsim} L_{d}^{i} X} \right) + \alpha_{2} Tr\left( {Z^{T} \mathop \sum \limits_{i = 1}^{nsim} L_{t}^{i} X^{T} } \right) } \\ \end{array}$$

In Eq. (18), $\alpha_{1} > 0$ and $\alpha_{2} > 0$ are the balancing parameters, ${\text{Tr}}\left( . \right)$ is the trace of a matrix, nsim indicates the number of similarity matrices (similar reference [11] nsim = 5). L_d_ and L_t_ are the graph Laplacians [72] for S_d_ and S_t_, where ${\text{L}}_{{\text{d}}} = {\text{D}}_{{\text{d}}} - {\text{S}}_{{\text{d}}}$ and ${\text{L}}_{{\text{t}}} = {\text{D}}_{{\text{t}}} - {\text{S}}_{{\text{t}}}$ are computed, respectively. D_d_ and D_t_ are degree matrices for drugs and targets that are calculated as ${\text{D}}_{{\text{d}}}^{{{\text{ii}}}} = \mathop \sum \limits_{{\text{j}}} {\text{S}}_{{\text{d}}}^{{{\text{ij}}}}$ and ${\text{D}}_{{\text{t}}}^{{{\text{ii}}}} = \mathop \sum \limits_{{\text{j}}} {\text{S}}_{{\text{t}}}^{{{\text{ij}}}}$.

As shown in Algorithm 2, a method proposed by Mongia et al. [11] is used to solve this equation. It should be noted that this algorithm uses the RRSVD_DTI method that is proposed for DTI prediction in this paper.
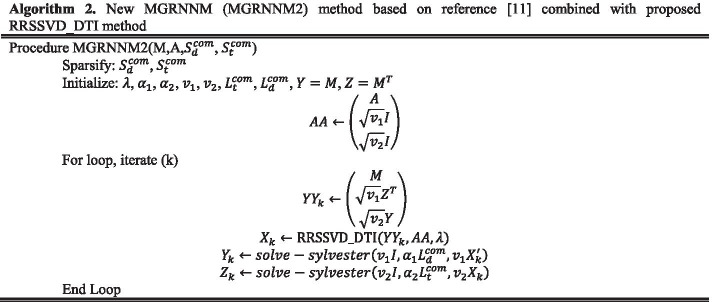


In Algorithm 2, $S_{d}^{com} = S_{d} + S_{d}^{cos} + S_{d}^{cor} + S_{d}^{ham} + S_{d}^{jac} = \mathop \sum \limits_{i = 1}^{nsim} S_{d}^{i}$ and $S_{t}^{com} = S_{t} + S_{t}^{cos} + S_{t}^{cor} + S_{t}^{ham} + S_{t}^{jac} = \mathop \sum \limits_{i = 1}^{nsim} S_{t}^{i}$ represent the combined similarity for drug and target, $D_{d}^{com} = diag\left( {\mathop \sum \limits_{j} S_{d}^{Com} } \right)$ and $D_{t}^{com} = diag\left( {\mathop \sum \limits_{j} S_{t}^{Com} } \right)$ represent the combined degree matrix for the drug and target also $L_{d}^{com} = D_{d}^{com} - D_{d}^{com}$ and $L_{t}^{com} = D_{t}^{com} - D_{t}^{com}$ represent the combined Laplacian matrix for the drug and target, respectively.

### Result

In this section, the experiments and results based on the proposed method are analyzed separately. In the first step, datasets and how to divide them into training and testing sets are presented. All experiments and extracted parameters are performed separately for each dataset under different validation settings. The following compares the proposed method with other new methods based on AUC, AUPR and time criteria.

### Dataset description

Reference [14] examines information on drug and target proteins interactions for the public databases; KEGG BRITE [73], BRENDA [74] SuperTarget [75] and DrugBank [76]. In this paper, similar to [2, 11, 14], four benchmark datasets are used, which are from four different classes of target proteins. In fact, these benchmarks are simulated from public databases. The following is a description of these datasets:

- **Enzymes (Es):** In this dataset, 445 drugs, 664 targets and 2926 interactions have been extracted.
- **Ion channels (ICs):** In this dataset, 201 drugs, 204 targets and 1476 interactions have been extracted
- **G protein-coupled receptors (GPCRs):** In this dataset, 223 drugs, 95 targets and 635 interactions were extracted
- **Nuclear receptors (NRs):** In this dataset, 54 drugs, 26 targets and 90 interactions have been extracted

It should be noted that these datasets are simulated from public databases which at the link: http://web.kuicr.kyoto-u.ac.jp/supp/yoshi/drugtarget/ are publicly available.

### Experimental setup

In this section, the setting of datasets is based on recent work done on the DTI problem. Three cross-validation settings (CVS) as named CVS1, CVS2 and CVS3 are introduced [6]. In CVS1, standard setting for evaluation, the target-drug pairs for the test set were randomly selected for prediction. In CVS2 and CVS3, settings are performed to evaluate the ability of methods to predict interactions for novel drugs (i.e., drugs for which no interaction information is available) and novel targets, respectively. It can be pointed out that in CVS2, entire drug profiles and CVS3, total target profiles are selected as a test set.

When at least one DTI is known for d_i_ and t_j_ respectively in the training data the CVS1 predicts the unknown pair (d_i_, t_j_). To prevent using the pairs, CV used the pairs between the drugs having at least 2 targets and the targets interacting with at least 2 drugs, which should be used in three other scenarios. Some of these pairs are selected randomly for testing in each round of CV and the union of the rest of them and other entries are used for training.

However, when there are no DTIs for observation of new drugs and new targets in the training data, CVS2 and CVS3 predict new drugs and new targets respectively.

Performance of CV on drugs in CSV2, where the rows corresponding to drugs are randomly blinded for testing and the remaining rows are used for training. Performance of CV on targets in CSV3, where the columns (accounting for targets) are randomly blinded for testing and the resting columns are used for training.

**Fig. 1:**
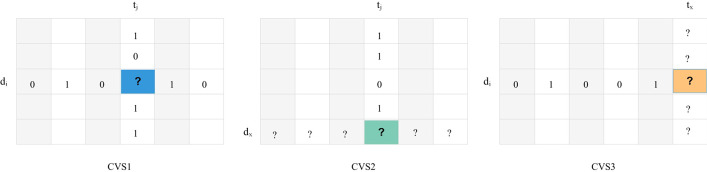
Presentation of cross-validation schemes for three scenarios. Each column represents a scenario. Row includes the DTI matrices, in which the entries marked with “?” are the pairs of interest to be tested

We have made various tasks of CV under 3 scenarios shown in Fig. 1, respectively [28].

Similarly to the method presented by Mongia et al. [11], tenfold cross-validation (CV) is used, where data was divided into tenfolds and out of those 10 folds, one was selected as a test set while the remaining ninefolds were considered as a training set. In experiments, 5 repetitions of tenfold CV for each of the methods under three CVS are performed. For a more accurate evaluation, in each repetition, all CVSs for each dataset are similar to Mongia and Ezzat study [6, 11].

### Evaluation metrics

To evaluate the performance of the proposed method, Area Under the ROC Curve (AUC) and Area Under the Precision-Recall curve (AUPR) criteria based on Mongia and Ezzat study [6, 11] have been used. In the following, the requirements are introduced:

- AUC is a famous quality measure of ranking performance. It uses the ROC curve, which is a graphical plot that illustrates the diagnostic ability with a positive rate for a method as a function of the false-positive rate. AUC measures all two-dimensional areas below the ROC curve. It is also used as a measure of classification performance, aggregating over decision thresholds. Interpreting AUC shows a better model that is a random positive example more highly than a random negative example.
- AUPR is another measure which is used to evaluate the performance of DTI methods in this paper. It uses the precision–recall curve, which is a ratio of true positives plot that illustrates the positive predictions for each given recall rate. AUPR performance evaluation shows this area under the Precision-Recall curve punishes more false positives than AUC. The AUPR offers a quantitative assessment of the separation of true interactions from true non-interactions among predicted scores. For this reason, due to few true drug–target interactions, AUPR is a more important qualitative scale than AUC which finds true drug–target interactions among prediction scores.

### Parameter settings

In this paper, cross-validation on the training set is used to set the parameters of the proposed method. In fact, experiments are designed under each cross-validation setting to find the best parameter for each dataset. The considered parameters are $P, \lambda , \alpha_{1} , \alpha_{2} , v_{1} , v_{2} ,r$. It should be noted that the ranges for $\lambda , \alpha_{1} , \alpha_{2} , v_{1} , v_{2}$ and $P,r$ parameters are considered (0,1) and (1,10), respectively. All the extracted parameters are shown in Table 1.

**Table 1:** Extracted parameters for the proposed method and [11]

Validation setting	Datasets	Parameters						
		$P$	$\varvec{\lambda }$	$\alpha_{1}$	$\alpha_{2}$	$v_{1}$	$v_{2}$	$r$
CVS1	NR	2	0.1	0.1	0.01	0.1	0.1	4
	GPCR	2	0.5	0.5	0.1	0.1	0.5	4
	IC	5	0.1	0.01	0.1	0.1	0.01	4
	E	5	0.1	0.01	0.1	0.1	0.1	4
CVS2	NR	2	0.01	0.01	0.01	0.01	0.1	4
	GPCR	2	0.01	0.1	0.01	0.01	0.01	4
	IC	5	0.1	0.01	0.1	0.1	0.1	4
	E	5	0.01	0.1	0.01	0.1	0.01	4
CVS3	NR	2	0.01	0.01	0.01	0.01	0.1	4
	GPCR	2	0.1	0.01	0.01	0.1	0.1	4
	IC	2	0.1	0.01	0.1	0.01	0.1	4
	E	2	0.1	0.01	0.1	0.01	0.01	4

It is necessary to mention, implementations^1^ were performed in MATLAB programming language on hardware configuration, 4G memory, and Core i7 M620 2.6 GHz CPU.

### Interaction prediction

**Fig. 2:**
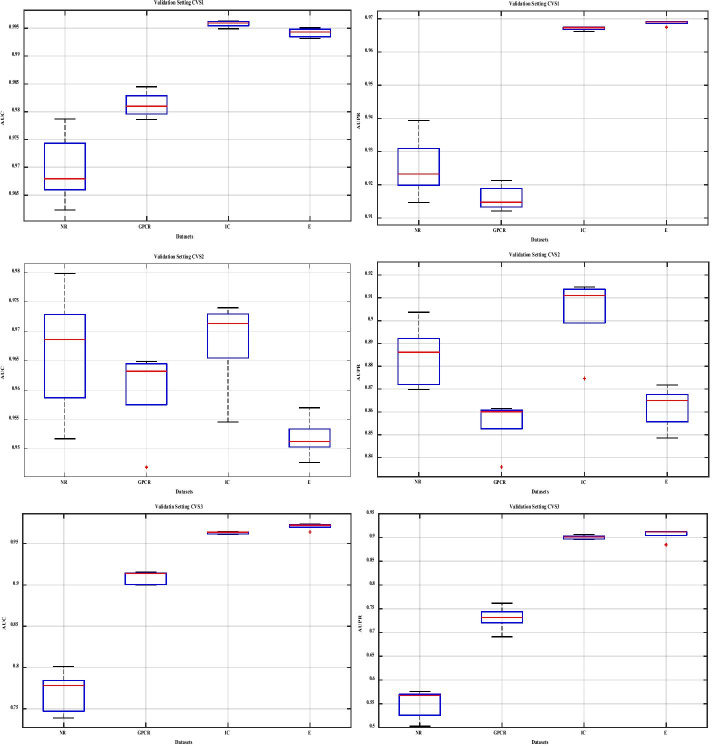
Boxplot diagram based on the proposed method in 5 times run under different validation settings. In each diagram, the results are reported on four datasets. The left column shows the AUC and the right column shows the AUPR. Each row from top to bottom shows the results under validation setting CVS1, validation setting CVS2 and validation setting CVS3, respectively

In Fig. 2, the results obtained in each validation set are shown by boxplot. In fact, this diagram is drawn based on five different runs on four databases. Two criteria, AUC and AUPR, have been considered in drawing this diagram. The results show the performance of the proposed method which is appropriate in each run and is without outliers.

### Comparison with the others methods

In this paper, to evaluate the proposed method, experiments have been designed to compare this method with 6 state-of-the-art methods introduced in recent years. In the following, we will describe these methods.

Weighted Graph Regularized Matrix Factorization (WGRMF) method [20] was introduced in 2016. In this method, since the data are located on or close to low-dimensional non-linear manifolds, two methods of matrix factorization are proposed, which in these methods, graph regularization is used. Also, a preprocessing step has been presented to improve the predictions of a “new drug” and a “new target” by introducing intermediate interaction likelihood scores.

In 2019, an improved graph regularized matrix factorization (GRMF) method was proposed to learn DTI flow patterns by combining the matrix analysis method called L_1,2_-GRMF [77]. In this method, WKNKN for preprocessing is used to improve prediction accuracy.

The Collaborative Matrix Factorization (CMF) method [35] was introduced in 2013. The main idea of this method is to use more than one target and drug similarity matrix. In this method, a weighted matrix for the automatic selection of similarities is estimated to improve DTI prediction.

Subsequently, a factor model called Multiple Similarities Collaborative Matrix Factorization (MSCMF) is proposed in which drugs and targets are projected in a common low-rank feature space. Finally, these two low-rank matrices and weights associated with similarity matrices are estimated by an alternating least squares algorithm.

Regularized Least Square Weighted Nearest Neighbor profile (RLS-WNN) method [25] was introduced in 2013. In this method, a simple weighted nearest neighbor procedure is introduced which is claimed that the procedure has performed well in DTI prediction, and to improve on previous work, this procedure is combined with the recent machine learning method.

Multi Graph Regularized Nuclear Norm Minimization (MGRNNM) method [11] was proposed in 2020. In this method, a new framework for predicting DTI from three inputs; known drug–target interaction network, similarities over drugs and those over targets is proposed. A method for finding a low-rank interaction matrix has been introduced. This matrix is made up of graphs that represent the proximities of drugs and targets. This paper proposes to capture the proximities exhaustively in predicting DTI, various multiple drug-drug similarities and target-target similarities as multiple graph Laplacian (over drugs / targets) regularization terms be used.

Four references are introduced specifically for the DTI prediction task. In the following, two references are introduced as baseline references.

Matrix completion (MC) method [78] was introduced in 2011. The paper focuses on solving matrix completion problems. In this regard, a non-convex optimization problem is proposed to solve the matrix completion. The proposed method is a variant of convex nuclear-norm minimization, with a fast numerical algorithm to solve it.

Matrix Completion on Graphs (MCG) method [79] was introduced in 2014. This paper introduces a novel matrix completion model for several real-world applications such as recommender systems. In this new model, the proximity information is used. It is stated that the purpose of this method is to find a low-rank solution created by the proximities of rows and columns. It should be noted that these proximities are encoded by graphs.

Neighborhood Regularized Logistic Matrix Factorization (NRLMF) method [36] was introduced in 2016. This method is based on the possibility of interacting a drug with a target through the logistic matrix factorization. NRLMF is more important in drug–target interaction pairs (positive observations) than in unknown pairs (negative observations). Because positive observations have already been experimentally confirmed, they are usually more reliable. For this reason, the local structure of drug–target interaction data has also been used through neighborhood adjustment to achieve better predictive accuracy.

DDR method was introduced in 2018 [80]. DDR works by using multiple similarities between drugs and considerable similarities between target proteins through a heterogeneous graph containing known DTIs.

Triple Matrix Factorization-based model (TMF) [28] was introduced in 2018. This model shows a new sight for the effective mechanism of DTIs by indicating prevailing features. TMF assesses the predictions on four benchmark datasets over different screening scenarios which represent its considerable priority.

In this section, a comparison between the proposed method and the current prediction methods is performed. AUC and AUPR criteria were used to evaluate the performance. Tables 2, 3, 4 and 6 show the results found on AUPR under validation setting; CVS1, CVS1 and CVS1 in four data sets, respectively. Tables 3, 5, 6 and 7 also show the results based on AUC under validation setting; CVS1, CVS1 and CVS1 in four datasets, respectively. In these tables, the best result are shown in bold. As shown in the tables, the proposed method performed well in all four data sets and all three validation sets. It should be noted that the SMGRNNM method is the same method presented in the Mongia et al. study [11], which uses only the standard similarity matrices.

**Table 2:** AUPR results for interaction prediction under validation setting CVS1

Method	Datasets			
	E	IC	GPCR	NR
MGRNNM [11]	0.9660 (0.0006)	0.9585 (0.0013)	0.8515 (0.0033)	0.8791 (0.0019)
SMGRNNM [11]	0.9014 (0.0018)	0.9298 (0.0026)	0.7483 (0.0039)	0.6408 (0.0234)
MC[78]	0.7882 (0.0022)	0.8868 (0.0028)	0.6481 (0.0116)	0.3950 (0.0298)
MCG[79]	0.7621 (0.0025)	0.8346 (0.0025)	0.5956 (0.0102)	0.4558 (0.0202)
WGRMF [20]	0.8768 (0.0020)	0.9225 (0.0022)	0.7370 (0.0024)	0.6016 (0.0378)
WKNKN + L_1,2_-GRMF [77]	–	–	–	–
RLS-WNN[25]	0.8093 (0.0045)	0.8459 (0.0106)	0.6933 (0.0226)	0.7072 (0.0290)
CMF [35]	0.8837 (0.0026)	0.9373 (0.0019)	0.7543 (0.0017)	0.6383 (0.0149)
NRLMF [36]	0.892 (0.006)	0.906 (0.008)	0.749 (0.015)	0.728 (0.041)
TMF [28]	0.952 (0.002)	0.952 (0.002)	0.844 (0.006)	0.811 (0.035)
DDR [80]	0.92	0.92	0.79	0.83
Proposed method	**0.9687 (0.0007)**	**0.9670 (0.0005)**	**0.9159 (0.0036)**	**0.9253 (0.0091)**

**Table 3:** AUC results for interaction prediction under validation setting CVS1

Method	Datasets			
	E	IC	GPCR	NR
MGRNNM [11]	**0.9955 (0.0003)**	0.9947 (0.0004)	0.9785 (0.0020)	0.9660 (0.0056)
SMGRNNM [11]	0.9798 (0.0004)	0.9829 (0.0012)	0.9531 (0.0028)	0.9083 (0.0058)
MC [78]	0.9596 (0.0015)	0.9415 (0.0015)	0.8110 (0.0055)	0.5882 (0.0253)
MCG [79]	0.8753 (0.0023)	0.9539 (0.0010)	0.8977 (0.0047)	0.8315 (0.0165)
WGRMF [20]	0.9647 (0.0013)	0.9747 (0.0022)	0.9432 (0.0010)	0.8892 (0.0153)
WKNKN + L_1,2_-GRMF [77]	–	–	–	–
RLS-WNN [25]	0.9635 (0.0014)	0.9786 (0.0026)	0.9458 (0.0044)	0.9329 (0.0114)
CMF [35]	0.9705 (0.0013)	0.9832 (0.0008)	0.9493 (0.0031)	0.8679 (0.0124)
NRLMF [36]	0.987 (0.001)	0.989 (0.001)	0.969 (0.004)	0.950 (0.011)
TMF [28]	0.989 (0.001)	0.989 (0.001)	0.9830 (0.003)	**0.978 (0.008)**
DDR [80]	0.97	0.98	0.96	0.92
Proposed method	0.9941 (0.0008)	**0.9958 (0.0005)**	**0.9832 (0.0022)**	0.9698 (0.0062)

**Table 4:** AUPR results for interaction prediction under validation setting CVS2

Method	Datasets			
	E	IC	GPCR	NR
MGRNNM [11]	0.8603 (0.0095)	0.9026 (0.0197)	0.8538 (0.0112)	0.8773 (0.0125)
SMGRNNM [11]	0.4089 (0.0104)	0.3650 (0.0178)	0.4175 (0.0076)	0.5620 (0.0262)
MC[78]	0.0114 (0.0005)	0.0473 (0.0035)	0.0404 (0.0017)	0.1120 (0.0206)
MCG[79]	0.0457 (0.0008)	0.0925 (0.0013)	0.1091 (0.0044)	0.2404 (0.0337)
WGRMF [20]	0.4019 (0.0128)	0.3666 (0.0169)	0.4247 (0.0113)	0.5695 (0.0136)
WKNKN + L_1,2_-GRMF [77]	0.386 (0.013)	0.356 (0.0012)	0.394 (0.007)	0.573 (0.011)
RLS-WNN[25]	0.2409 (00.272)	0.3090 (0.0200)	0.3463 (0.0106)	0.5373(0.0216)
CMF [35]	0.3848 (0.0094)	0.3538 (0.0137)	0.4059 (0.0104)	0.5203 (0.0250)
NRLMF [36]	0.358 (0.040)	0.344 (0.033)	0.364 (0.023)	0.545 (0.054)
TMF [28]	0.438 (0.016)	0.376 (0.017)	0.428 (0.011)	0.541 (0.033)
DDR [80]	0.73	0.69	0.63	0.71
Proposed method	**0.8619 (0.0089)**	**0.9042 (0.0167)**	**0.8552 (0.109)**	**0.8841 (0.0135)**

**Table 5:** AUC results for interaction prediction under validation setting CVS2

Method	Datasets			
	E	IC	GPCR	NR
MGRNNM [11]	0.9460 (0.0033)	**0.9714 (0.0095)**	0.9567 (0.0084)	0.9533 (0.0127)
SMGRNNM [11]	0.8260 (0.0108)	0.7913 (0.0090)	0.8805 (0.0024)	0.8452 (0.0215)
MC [78]	0.5060 (0.0090)	0.5512 (0.0034)	0.5855 (0.0039)	0.5294 (0.0200)
MCG [79]	0.7413 (0.0118)	0.7196 (0.0071)	0.7745 (0.0027)	0.6992 (0.0244)
WGRMF [20]	0.7982 (0.0144)	0.7902 (0.0149)	0.8800 (0.0025)	0.8615 (0.0244)
WKNKN + L_1,2_-GRMF [77]	–	–	–	–
RLS-WNN [25]	0.7755 (0.0093)	0.7669 (0.0140)	0.8524 (0.0072)	0.8390 (0.0261)
CMF [35]	0.7952 (0.0110)	0.7576 (0.0125)	0.8067 (0.0067)	0.8124 (0.0228)
NRLMF [36]	0.871 (0.017)	0.813 (0.027)	0.895 (0.011)	0.900 (0.021)
TMF [28]	0.843 (0.012)	0.819 (0.011)	0.882 (0.009)	0.886 (0.017)
DDR [80]	0.84	0.94	0.91	0.90
Proposed method	**0.9518 (0.0033)**	0.9682 (0.0078)	**0.9600 (0.0075)**	**0.9663 (0.105)**

**Table 6:** AUPR results for interaction prediction under validation setting CVS3

Method	Datasets			
	E	IC	GPCR	NR
MGRNNM [11]	0.9041 (0.0125)	0.9029 (0.0024)	0.7228 (0.0323)	0.5418 (0.0309)
SMGRNNM [11]	0.8087 (0.0156)	0.8079 (0.0096)	0.5963 (0.0336)	0.4356 (0.0177)
MC[78]	0.0124 (0.0005)	0.0421 (0.0043)	0.0549 (0.0105)	0.0850 (0.0227)
MCG[79]	0.0691 (0.0009)	0.2256 (0.0038)	0.1061 (0.0027)	0.2669 (0.0288)
WGRMF [20]	0.8070 (0.0185)	0.8128 (0.0069)	0.6093 (0.0314)	0.4643 (0.0183)
WKNKN + L_1,2_-GRMF [77]	0.799 (0.016)	0.826 (0.008)	0.617 (0.024)	0.519 (0.038)
RLS-WNN[25]	0.5465 (0.0144)	0.7437 (0.0088)	0.5397 (0.0193)	0.4907 (0.0326)
CMF [35]	0.7808 (0.0131)	0.7786 (0.0108)	0.5989 (0.0323)	0.4774 (0.0173)
NRLMF [36]	0.812 (0.018)	0.785 (0.028)	0.556 (0.038)	0.449 (0.079)
TMF [28]	0.866 (0.007)	0.853 (0.008)	0.677 (0.028)	**0.675 (0.062)**
DDR [80]	0.82	0.80	0.61	0.64
Proposed Method	**0.9063 (0.01**20)	**0.9006 (0.0040)**	**0.7306 (0.0254)**	0.5496 (0.0307)

**Table 7:** AUC results for interaction prediction under validation setting CVS3

Method	Datasets			
	E	IC	GPCR	NR
MGRNNM [11]	0.9683 (0.0043)	0.9541 (0.0019)	0.8975 (0.0093)	0.7502 (0.0285)
SMGRNNM [11]	0.9246 (0.0091)	0.9346 (0.0041)	0.8798 (0.0134)	0.7263 (0.0211)
MC[78]	0.5234 (0.0057)	0.4724 (0.0065)	0.5683 (0.0310)	0.3767 (0.0204)
MCG[79]	0.8065 (0.0012)	0.7871 (0.0069)	0.6289 (0.0151)	0.6522 (0.0297)
WGRMF [20]	0.9338 (0.0071)	0.9460 (0.0034)	0.8892 (0.0110)	0.7967 (0.0132)
WKNKN + L_1,2_-GRMF [77]	–	–	–	–
RLS-WNN[25]	0.9067 (0.0105)	0.9286 (0.0046)	0.8694 (0.0146)	0.8124 (0.0202)
CMF [35]	0.9272 (0.0050)	0.9368 (0.0032)	0.8966 (0.0073)	0.8373 (0.0083)
NRLMF [36]	0.966 (0.005)	0.964 (0.007)	0.930 (0.012)	0.851 (0.027)
TMF [28]	0.976 (0.001)	0.972 (0.002)	**0.959 (0.007)**	**0.929 (0.029)**
DDR [80]	0.92	0.97	0.93	0.88
Proposed method	**0.9770 (0.0038)**	**0.9830 (0.0013)**	0.9087 (0.0075)	0.7694 (0.0249)

### Time complexity

In this section, the time complexity of the proposed algorithm is compared with MGRNNM method [11] as one of the best methods presented in DTI. Since Algorithm 2 is an iterative solution, in this paper, similar to MGRNNM, the time complexity is calculated for each iteration. In the proposed method and MGRNNM, in each iteration two.

Sylvester equations and one NNM are solved. According to Kirrinnis et al. study [81], the complexity of solving the Sylvester equation is equal to $O\left( {n^{b} \log \left( n \right)} \right)$, in which the parameter b is between 2 and 3. To solve NNM in MGRNNM, the SVS method is used which the complexity of this algorithm is equal to $O\left( {n^{3} } \right)$ in each iteration. In fact, MGRNNM has time complexity in each iteration $O\left( {n^{3} + n^{b} \log \left( n \right)} \right)$. The proposed method uses the RRSSVD_DTI algorithm to solve NNM. According to Hastie et al. study [68], this algorithm requires $O\left( {r^{3} } \right)$ time per each iteration. It can be said that the time complexity of the proposed algorithm in each iteration is equal to $O\left( {r^{3} + n^{b} \log \left( n \right)} \right)$ and since $r \ll {\text{min}}\left( {m,n} \right)$, the time complexity of the proposed algorithm is minor than MGRNNM.

**Fig. 3:**
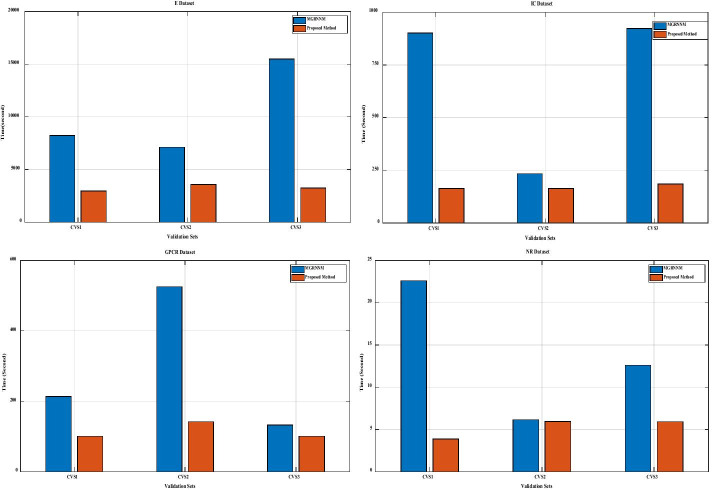
Comparing the running time of the proposed method with Mongia et al. study [11] in four datasets. In each bar chart, the blue color shows the running time of Mongia et al. study [11] and the orange color indicates the running time of the proposed method. The top row shows the running time in the E and IC datasets, respectively, from left to right. The bottom row shows the running time in the GPCR and NR datasets, respectively, from left to right

In the following, more details of the time complexity are presented. The proposed method and MGRNNM are compared on four datasets based on running time. As shown in Fig. 3, the proposed method performed well on all the datasets.

It should be noted that in these applications, the online web detection system is designed to meet the needs of many people simultaneously. In such scenarios, response time can be very significant, which shows the proper performance of the proposed method in time complexity that our approach can be used in DTI.

## Discussion

The use of adjacency matrices to represent the interaction between drug and target has been considered by many researchers in recent years. In DTI prediction, the detection of low-rank interaction has been significant. Using LRMA methods to solve these problems can improve performance. The results reported in this article showed that unifying matrix factorization and nuclear norm minimization approaches based on similarity matrix has a good effect on solving low-rank problem.

AUC, AUPR and running time are commonly used to evaluate the performance of methods in DTI. It should be noted that the use of a graph to express the adjacency of the target and the drug in this application has improved the performance of the proposed method. Overall, this paper presents a powerful and fast method for applying DTI, which shows improved performance in four benchmark datasets.

To evaluate the proposed method, all four datasets are divided into three cross-validation settings. Results were reported as mean and variance of AUC and AUPR per 5 runs. It should be noted that the proposed method is compared with several methods [11, 20, 25, 28, 35, 36, 78–80]. The results have been shown the proposed method which was based on the similarity matrix, had the best performance. The time complexity of the proposed method was more appropriate than other methods.

## Conclusion

In drug-related processes such as drug discovery, drug side-effect prediction and drug repurposing, the interaction between drugs and targets (proteins) is very important. Drugs effect on targets (proteins) by altering the pharmaceutical functions of targets, such as enzymes, ion channels, G protein-coupled receptors (GPCRs), and nuclear receptors. DTIs analysis requires costly and time-consuming experiments. In this regard, CP-based approaches have been used to narrow down the search space and also reduce the cost and time of experiments.

In this paper, CP based on chemogenomic methods in DTI is used. It was shown that the use of similarity matrices in this application provides the best performance compared to the other methods. Also, we presented that the use of unifying of graph regularized nuclear norm with bilinear factorization can be very effective in predicting DTI. In this paper, the proposed method on four datasets based on three different cross-validation settings is compared with six state-of-the-art methods. The results show a better performance of the proposed method. In general, the superiority of the proposed method can be expressed as follows:

- There is a trade-off parameter between the loss function and the low-rank regularization as λ in the NNM approach which is induced by the nuclear norm. The use of low-rank priors to many applications has been developed by these models where Z (in Eq. (3)) is low rank but its rank is not known a priori. These models also have multiple problems despite their convexity and theoretical guidelines for the choice of λ [9]. First, it is unclear how to impose a certain rank in Z: adjusting λ so Z has a predetermined rank usually produces unpleasant results than imposing it directly in Eq. (3) in many works. Second, it is impossible to obtain the Z factorization in Eq. (7) Causes not use the “kernel trick”. Third, Eq. (7) is a Semidefinite Program (SDP). Off-theshelf SDP optimizers just divide into hundreds of variables, not amenable to the high dimensionality typically found in DTI problems. While many studies improve this issue, they still perform an SVD of Z in each iteration and make them disproportionate for managing dense and large-scale datasets. This paper indicates many nuclear norms regularized problems of the form (7) which can be optimized with a bilinear factorization of Z = UV^T^ by using the variational definition of the nuclear norm. In this paper, a unification of traditional bilinear factorization and nuclear norm approaches under one formulation in DTI applications have been proposed. Based on this result, we can analyze the conditions that both methods are equal and offer the best solution when they are not. This article explains how the proposed method can be used in DTI application. In the reference [9], the method based on nuclear norm regularization has been used. The optimization equations of the proposed method and its solution are expressed differently.
- Unifying nuclear norm with bilinear factorization is presented based on the similarity of drug-drug and target-target, which has caused the advantages of both methods to be combined.
- Rank-Restricted Soft Singular Value Decomposition method is used to optimize the nuclear norm minimization in the DTI problem. This method has not been used in this application so far. It was shown that the use of this method could have appropriate performance in data based on graph similarity.
- One of the critical parameters in evaluating the performance of these methods is the running time. With the increasing growth of this data, the use of computational methods will increase; on the other hand more similarity measures and samples with more features can be used. The time complexity of the running time is $O\left( {r^{3} + n^{b} \log \left( n \right)} \right)$ for each iteration in the proposed algorithm. This complexity is polynomial, which has performed better than other new methods.
- The performance of the proposed method based on the AUC and AUPR measures had the best performance and also the results were suitable in datasets with different features.
- The proposed method can be widely used in other applications of CP. Using the proposed method does not require a complex process.

## Data Availability

Datasets are simulated from public databases which at the link: http://web.kuicr.kyoto-u.ac.jp/supp/yoshi/drugtarget/ are publicly available. The authors declare that they have provided the code and data publicly accessible, which can be downloaded from: https://github.com/ali289/DTI-GRNNwBF/

## Abbreviations

DTIs: Drug–target interactions
RRSSVD: Rank-Restricted Soft Singular Value Decomposition
E: Enzymes
ICs: Ion channels
GPCRs: G protein-coupled receptors
NRs: Nuclear receptors
AUC: Area Under the ROC Curve
AUPR: Area Under the Precision-Recall curve
CP: Computational prediction
NNM: Nuclear norm minimization
SVs: Singular values
LRMF: Low rank matrix factorization
SVD: Singular Value Decomposition
LS: Least Squares
NNP: Nuclear norm proximal
CVS: Cross-validation settings
CV: Cross-validation
WGRMF: Weighted Graph Regularized Matrix Factorization
CMF: Matrix Factorization
MSCMF: Multiple Similarities Collaborative Matrix Factorization
RLS-WNN: Regularized Least square nearest neighbor profile
MGRNNM: Multi Graph Regularized Nuclear Norm Minimization
MC: Matrix completion
MCG: Matrix completion on graphs, NRLMF: Neighborhood Regularized Logistic Matrix Factorization

## Footnotes

1 https://github.com/ali289/DTI-GRNNwBF/
